# Comparative study of oral iron and intravenous iron therapy in pregnancy induced anemia

**DOI:** 10.6026/973206300221655

**Published:** 2026-03-31

**Authors:** Shrutika S Khapre, Sabita Yograj

**Affiliations:** 1 Department of Obstetrics and Gynaecology, Datta Meghe Institute of Higher Education and Research (DMIHER), Sawangi (Meghe), Wardha, Maharashtra, India; 2 Department of Physiology, Coordinator (MEU & CC), Ex-Dean (Academics), Shri Mata Vaishno Devi Institute of Medical Excellence (SMVDIME), Kakryal, Jammu and Kashmir, India

**Keywords:** Pregnancy induced anemia, iron deficiency anemia, oral iron therapy, intravenous iron therapy, hemoglobin

## Abstract

Pregnancy-induced anemia, primarily caused by iron deficiency, poses significant risks to maternal and fetal health, especially in developing countries. Oral iron therapy is commonly used but has limitations due to poor compliance and gastrointestinal side effects. Therefore, it is of interest to compare the efficacy and tolerability of oral versus intravenous iron therapy in pregnant women with iron deficiency anemia. Results show that intravenous iron significantly improves hemoglobin levels more effectively and with fewer adverse effects than oral iron. This study advances knowledge by highlighting intravenous iron as a superior treatment option for rapid correction of pregnancy-induced anemia.

## Background:

Anemia in pregnancy remains one of the most prevalent and significant public health challenges worldwide, particularly in low and middle-income countries. It is defined as a reduction in the oxygen-carrying capacity of blood, most commonly due to decreased hemoglobin concentration and is associated with adverse maternal and fetal outcomes [1]. According to the World Health Organization, anemia affects nearly 40% of pregnant women globally, with iron deficiency accounting for the majority of cases. In countries such as India, the burden is even higher due to nutritional deficiencies, frequent pregnancies, poor socioeconomic conditions and limited access to healthcare. Pregnancy induced anemia, therefore, represents not only a medical concern but also a social and economic problem that requires effective and timely intervention [2]. During pregnancy, iron requirements increase substantially to support the expansion of maternal red blood cell mass, development of the fetus and placenta and compensation for blood loss at delivery. The total iron requirement during pregnancy is estimated to be approximately 1000 mg, which cannot be met by diet alone in most women. When this increased demand is not fulfilled, iron stores become depleted, leading to iron deficiency anemia. Pregnancy induced anemia is associated with fatigue, reduced work capacity, increased susceptibility to infections and impaired quality of life in the mother [3]. More importantly, it increases the risk of preterm birth, low birth weight, intrauterine growth restriction and perinatal mortality. Severe anemia also contributes significantly to maternal morbidity and mortality, particularly due to cardiac failure and hemorrhage during labor and the postpartum period [4]. Oral iron therapy has traditionally been the first-line treatment for pregnancy induced anemia because it is inexpensive, widely available and easy to administer. Oral iron preparations, usually in the form of ferrous salts, are effective in increasing hemoglobin levels in mild to moderate anemia when compliance is adequate. However, oral iron therapy is often associated with gastrointestinal side effects such as nausea, vomiting, epigastric discomfort, constipation and diarrhea [5]. These adverse effects frequently lead to poor adherence, especially in pregnant women who may already experience nausea and vomiting due to pregnancy itself. In addition, oral iron has limited absorption, which can be further reduced by concomitant intake of food, antacids, or underlying gastrointestinal disorders. As a result, the response to oral iron therapy is often slow and suboptimal, particularly in women with moderate to severe anemia or in those presenting late in pregnancy [6].

Intravenous iron therapy has emerged as an effective alternative to oral iron, especially in cases where rapid correction of anemia is required or when oral iron is poorly tolerated or ineffective. Modern intravenous iron formulations allow the administration of larger doses of iron in a shorter period of time, leading to a more rapid rise in hemoglobin levels and replenishment of iron stores [7]. This is particularly advantageous in the second and third trimesters of pregnancy, when time is limited and the consequences of untreated anemia can be severe. Intravenous iron bypasses the gastrointestinal tract, thereby avoiding absorption-related issues and improving treatment compliance [8]. Despite these advantages, intravenous iron therapy has historically been underutilized due to concerns regarding safety, cost and the need for hospital-based administration. Earlier formulations were associated with a higher risk of allergic reactions and anaphylaxis, which created hesitation among clinicians [9]. However, newer intravenous iron preparations have demonstrated improved safety profiles with minimal serious adverse effects when administered appropriately. Even so, the higher cost compared to oral iron and the requirement for trained personnel and monitoring facilities remain potential barriers, particularly in resource-limited settings [10]. Given these contrasting characteristics, there is ongoing debate regarding the optimal modality of iron supplementation for pregnancy induced anemia. While oral iron remains suitable for mild anemia and preventive supplementation, intravenous iron may offer superior efficacy in achieving faster hematological improvement in moderate to severe anemia [11]. Comparative evaluation of oral and intravenous iron therapy is therefore essential to guide clinical decision-making, optimize maternal and fetal outcomes and ensure rational use of healthcare resources. Factors such as the degree of anemia, gestational age, side-effect profile, patient compliance, cost-effectiveness and speed of hemoglobin correction must all be considered when selecting the appropriate treatment strategy [12]. In regions with a high prevalence of pregnancy induced anemia, standardized treatment protocols based on robust clinical evidence are crucial. Comparative studies provide valuable insights into the relative effectiveness, safety and acceptability of different treatment modalities [13]. By directly comparing oral iron and intravenous iron therapy in pregnant women with anemia, clinicians can better understand which approach yields superior outcomes under specific clinical circumstances. Such evidence is particularly relevant in tertiary care settings, where women often present with moderate to severe anemia and limited time remains before delivery. In view of the high burden of pregnancy induced anemia, its serious consequences for both mother and fetus and the limitations associated with conventional oral iron therapy, it is imperative to evaluate alternative treatment strategies [14]. Therefore, it is of interest to assess the relative efficacy, safety and feasibility of intravenous iron therapy compared to oral iron, especially in settings with high anemia prevalence, to guide clinical decision-making and improve maternal and fetal health outcomes.

## Methodology:

This original research was designed as a prospective, comparative, interventional study conducted in the Department of Obstetrics and Gynecology of a tertiary care teaching hospital over a defined 12-month period. After obtaining approval from the Institutional Ethics Committee, written informed consent was obtained from all participants prior to enrollment. A total of 100 pregnant women diagnosed with pregnancy-induced anemia were included in the study. The sample size was selected based on feasibility, patient availability during the study period and consistency with similar previously published comparative studies. Eligible participants were consecutively recruited from the antenatal outpatient department and inpatient wards until the required sample size was achieved. Inclusion criteria for the study were pregnant women aged 18-40 years, with a gestational age between 14 and 34 weeks, hemoglobin concentration between 7 g/dL and 10 g/dL and a diagnosis of iron deficiency anemia based on clinical and laboratory findings. All participants were required to be willing to participate and provide informed consent. Exclusion criteria included hemoglobin levels outside the range of 7-10 g/dL, anemia due to causes other than iron deficiency (*e.g.*, hemoglobinopathies, megaloblastic anemia), a history of hypersensitivity to iron preparations, chronic medical disorders such as renal disease, liver disease, or hematological disorders, multiple pregnancies, active infection, recent blood transfusion and women in the first trimester or beyond 34 weeks of gestation. The 100 participants were randomly allocated into two equal groups of 50 each using a simple randomization technique. Group A (Oral Iron Group) received oral iron therapy, while Group B (Intravenous Iron Group) received intravenous iron therapy. Participants in Group A received oral iron tablets containing 100 mg of elemental iron twice daily for 6 weeks and were counseled on proper intake, possible side effects and the importance of compliance. Participants in Group B received intravenous iron according to their calculated total iron requirement using standard dosing formulas. The intravenous iron was administered in divided doses under medical supervision, with monitoring for adverse reactions during and after infusion. Baseline data, including maternal age, parity, gestational age, socioeconomic status, dietary habits and baseline hemoglobin levels, were recorded at enrollment. Hemoglobin concentration was measured using an automated hematology analyzer at baseline and repeated after 6 weeks of therapy. Any adverse effects related to iron therapy were documented throughout the study period. The primary outcome measure was the rise in hemoglobin level after 6 weeks of treatment, while secondary outcome measures included treatment compliance, the incidence of adverse effects and the overall tolerability of oral versus intravenous iron therapy. Data were entered into a spreadsheet and analyzed using statistical software. Continuous variables were expressed as mean ± standard deviation and categorical variables were presented as frequencies and percentages. The paired t-test was used to compare pre- and post-treatment hemoglobin levels within groups and the unpaired t-test was used to compare outcomes between the two groups. A p-value of <0.05 was considered statistically significant.

## Results:

A total of 100 pregnant women with pregnancy-induced iron deficiency anemia were included in the final analysis, with 50 participants in each group (oral iron and intravenous iron). All enrolled participants completed the 6-week follow-up and their data were analyzed. The baseline demographic and clinical characteristics of both groups were comparable, with no statistically significant differences, indicating proper randomization. As shown in Table 1 (see PDF), both groups were statistically comparable at baseline. Both groups showed a significant rise in hemoglobin levels after 6 weeks of treatment; however, the increase was significantly higher in the intravenous iron group. The rise in hemoglobin was significantly greater in the intravenous iron group compared to the oral iron group (p < 0.001), as shown in Table 2 (see PDF). An independent t-test demonstrated a statistically significant difference in mean hemoglobin rise between the two groups. Table 3 (see PDF) confirms that intravenous iron therapy resulted in a significantly higher improvement in hemoglobin levels. Adverse effects were more commonly reported in the oral iron group, predominantly gastrointestinal symptoms, whereas intravenous iron therapy was well tolerated. As seen in Table 4 (see PDF), oral iron was associated with significantly higher gastrointestinal side effects. STATA version 16.0 was used for statistical analysis. The key findings from STATA are summarized in (Table 5 - see PDF). STATA analysis (Table 5 - see PDF) confirmed a statistically significant improvement in hemoglobin levels in both groups, with intravenous iron therapy showing superior efficacy. Overall, the results demonstrate that while both oral and intravenous iron therapies are effective in treating pregnancy-induced anemia, intravenous iron therapy leads to a faster and significantly greater rise in hemoglobin levels with better tolerability.

## Discussion:

In the present study of 100 pregnant women with iron deficiency anemia, both oral and intravenous iron therapies resulted in significant improvement in hemoglobin levels after six weeks, but the intravenous iron group showed a significantly greater rise in hemoglobin and better tolerability compared with oral iron. These findings are consistent with several previous studies in the literature. Lewkowitz *et al.* (2022) [15] conducted a randomized controlled trial comparing intravenous iron versus oral iron in pregnant women with iron deficiency anemia which were similar to our studies. Govindappagari and Burwick (2019) [16] performed a meta-analysis of 11 randomized controlled trials evaluating IV versus oral iron in pregnancy. Their analysis using STATA found that pregnant women receiving IV iron more often achieved target hemoglobin levels faster with fewer adverse reactions than those receiving oral iron, which corroborates our findings of more rapid hematological correction and better tolerability with intravenous therapy. Another earlier randomized trial by Khalafallah *et al.* (2010) [17] directly compared intravenous iron polymaltose with standard oral iron therapy in moderate iron deficiency anemia of pregnancy. In this study, the intravenous regimen produced significantly greater increases in hemoglobin and ferritin levels compared to oral iron alone, mirroring our observations of improved iron repletion and hematologic response with intravenous therapy. Further supporting evidence comes from Shafi *et al.* (2012) [18] who compared intravenous iron with oral iron in 200 pregnant women. Their results showed a significantly higher rise in hemoglobin and serum ferritin levels at 2, 4 and 6 weeks in the intravenous group, without any serious adverse events. This is in agreement with our study where intravenous iron improved iron stores and hemoglobin more effectively than oral iron. Overall, our findings reinforce the growing evidence base that intravenous iron therapy, when appropriately indicated, can achieve faster and more robust hematologic improvement with better tolerability compared to oral iron therapy in pregnancy-induced anemia. The consistent findings across individual RCTs and meta-analyses demonstrating higher hemoglobin increases, fewer side effects and at least equivalent safety profiles underscore the potential advantages of intravenous iron, particularly in women who have moderate to severe anemia, poor response to oral iron, or intolerance to oral therapy. However, despite clear hematologic benefits, some studies (*e.g.*, large meta-analyses) note that clinical outcomes such as the need for blood transfusion or neonatal outcomes may not always show statistically significant differences between the two treatment routes. This suggests that while intravenous iron improves laboratory parameters, further research is needed to assess long-term maternal and neonatal clinical outcomes, cost-effectiveness and optimal protocols for use in varied healthcare settings.

## Conclusion:

We show that both oral and intravenous iron therapies effectively improve hemoglobin levels in pregnancy-induced anemia, with intravenous iron leading to a faster and greater increase. Oral iron is suitable for mild anemia, while intravenous iron is more effective and better tolerated in moderate cases or when rapid correction is needed. Individualized treatment selection can optimize maternal outcomes in pregnancy-induced anemia.
